# Response to Nutrient Stress in the Industrial Model Bacterium *Cupriavidus necator*: A Thermal Proteome Profiling (TPP) Investigation

**DOI:** 10.1002/pmic.70045

**Published:** 2025-09-16

**Authors:** Kate McKeever, Jia‐Lynn Tham, Manuel Bruch, Tania Narancic, Kevin O’ Connor, Swathi Ramachandra Upadhya, Colm Ryan, Eugene T. Dillon, Kieran Wynne, Gerard Cagney

**Affiliations:** ^1^ BiOrbic – Bioeconomy Research Centre University College Dublin Dublin, Belfield Ireland; ^2^ UCD Conway Institute University College Dublin Dublin, Belfield Ireland; ^3^ School of Biomolecular and Biomedical Science University College Dublin Dublin, Belfield Ireland; ^4^ School of Computer Science University College Dublin Dublin, Belfield Ireland; ^5^ Systems Biology Ireland University College Dublin Dublin, Belfield Ireland

**Keywords:** *Cupriavidus necator*, expression proteomics, PHA biosynthesis, protein conformation, thermal proteome profiling

## Abstract

The facultative chemolithoautotroph *Cupriavidus necator* is capable of heterotrophic growth on diverse carbon sources, or of autotrophic growth using CO_2_ fixation with H_2_ as an energy source. Under stress conditions, it produces biodegradable polyesters (polyhydroxyalkanoates, PHAs) as a storage material occupying a high proportion of the total biomass. This metabolic versatility means that *C. necator* is under intense study for sustainable biotechnology processes; however, a relative lack of understanding of the overall regulatory architecture has limited its application. The major mechanisms by which proteins can respond to shifting cellular demands are protein expression change and/or allosteric regulation. Here, we use two powerful proteomics methods to investigate these responses in *C. necator* cells grown on balanced or low nitrogen (PHA‐inducing) media. Using quantitative proteomics and protein stability analysis (which can report on conformation change), we find that proteins across different pathways respond through one or both of these regulatory modes, including coordinated adaptation to nutrient stress by the PHA pathway, the Calvin cycle and ribosomal proteins. Overall, the study offers a valuable overview of global protein changes evoked by nutritional stress, and shows how the combined use of both proteomics approaches can identify key responsive proteins that would otherwise be undetected.

**Summary:**

- We report a comprehensive proteomics analysis of the important industrial bacterium *Cupriavidus necator*, using two state‐of‐the‐art approaches: expression proteomics and thermal proteome profiling.
- With intense interest worldwide in finding substitutes for petrochemical based plastics, organisms such as *C. necator* are under active investigation, since they produce a storage bioplastic material (PHA) and have a versatile metabolism including growth on carbon dioxide.
- To our knowledge, this is the first thermal proteome analysis of a lithoautotrophic organism. We compared global protein expression the under conditions that induce PHA production, and we analysed the thermal proteome under the same conditions.
- Each experiment yielded novel, interesting results pertinent to individual proteins or pathways; moreover, by combining both approaches, proteins regulated by expression change and/or conformation change were highlighted.

**Abbreviations**
CDW: cell dry weight
CETSA: cellular thermal shift assay
GCMS: gas chromatography‐mass spectrometry
LC‐MS: liquid chromatography‐mass spectrometry
LFQ: label‐free quantitation
PHA: polyhydroxyalkanoate
PHB: polyhydroxybutyrate
TPP: thermal proteome profiling

## 1 Introduction

**FIGURE 1:**
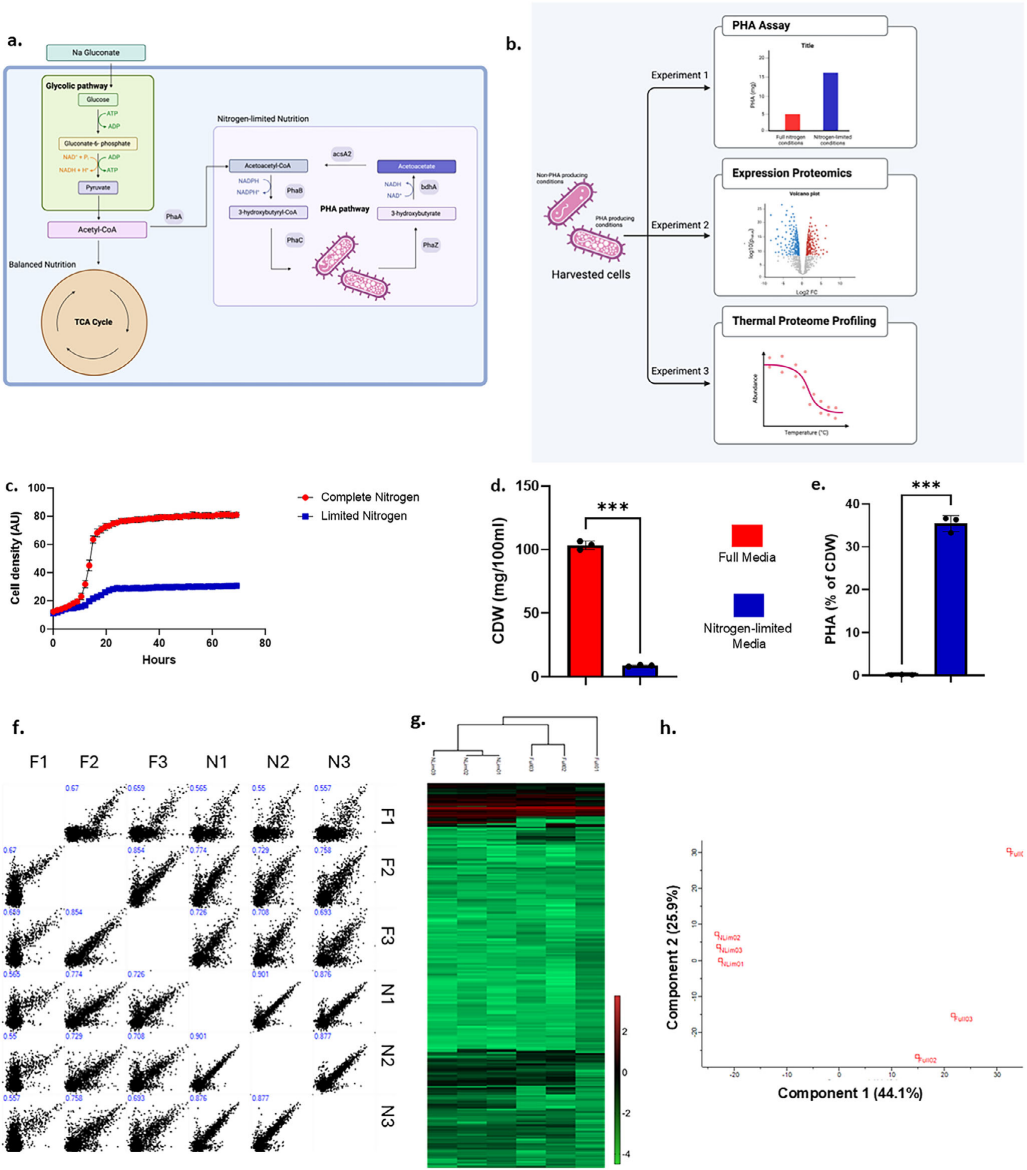
Investigating the nutritional stress response in *Cupriavidus necator*. (A) *C. necator* has versatile metabolic capabilities, with many potential substrates serving as carbon sources for heterotrophic growth. When nutritional resources are abundant, diversion of acetyl‐CoA into the TCA cycle is favoured, while under conditions of nutrient stress (e.g., low nitrogen, low phosphorus), diversion to PHB production pathways is favoured. (B) Overview of the experimental approach. *C. necator* cells were grown in complete media or in nitrogen‐limiting (PHA stimulating) media. The experimental model was validated (‘PHA Assay’) using gas chromatography‐mass spectrometry (GCMS) analysis of PHA content. Next, global liquid chromatography‐mass spectrometry (LC‐MS) analysis (‘expression proteomics’) was used to determine the relative abundance of individual proteins under the two experimental conditions. This was followed by an investigation of protein stability/conformation change under the same conditions using TMT labelling of samples subjected to a thermal gradient (‘thermal proteome profiling’). (C) Bacterial cells were grown in nitrogen‐limited (N‐lim) or balanced media (Full) conditions (*n* = 3 technical replicates). Cell growth was monitored by spectrophotometer (OD600nm). (D) Cell dry weight (CDW) was measured after 48 h growth (*n* = 3 technical replicates) (** unpaired Welch *t*‐test, *p* = 0.0009). (E) Polyhydroxyalkanoate (PHA) levels were monitored using gas chromatography‐mass spectrometry, GCMS (*n* = 3 technical replicates) (**** unpaired Welch *t*‐test, *p* = 0.0003). (F) Six samples (two growth media conditions, *n* = 3 technical replicates) were compared in a pairwise manner using Spearman's rank correlation coefficient (inset in blue). F indicates full media and N indicates nitrogen‐limited media; suffixes (−1,−2,−3) indicate replicate experiments. (G) A heat map of protein expression levels obtained using label‐free quantitation (LFQ) was analysed by hierarchical clustering. (H) Principal component analysis (PCA) of the six samples.

*Cupriavidus necator* is a bacterium with broad metabolic capacity that reflects its wide ecological distribution, which includes aquatic and soil environments, as well as industrial and polluted locations. Under balanced growth conditions, metabolic pathways optimal for cell growth are favoured, in particular diversion of acetyl‐CoA into the tricarboxylic acid (TCA) cycle. Under non‐balanced growth conditions, for example, where nitrogen or phosphorus are limiting, acetyl‐CoA may be diverted to an energy‐rich storage material within the polyhydroxyalkanoate (PHA) class: poly(3‐hydroxybutyrate), or polyhydroxybutyrate (PHB) [1]. Three enzymes acting on successive steps are critical for PHB production in *C. necator*: β‐ketothiolase (PhaA), which forms acetoacetyl‐CoA from two acetyl‐CoA molecules; NADPH‐dependent acetoacetyl‐CoA reductase (PhaB), which catalyses the production of 3‐hydroxybutyryl‐CoA from acetoacetyl‐CoA; and PHA synthase (PhaC), which produces PHA from 3‐hydroxybutyryl‐CoA monomers (Figure 1A). A depolymerase, PhaZ, can reverse this reaction to form 3‐hydroxybutyrate molecules in what has been viewed as a cyclic process to optimise the level of PHA in the cell according to metabolic requirements of the bacterium [2]. The PHA is stored intracellularly within hydrophobic inclusions (termed PHA granules, or carbonosomes) and is associated with proteins termed phasins, which are of unclear function but which demonstrate a carbonosome‐related phenotype upon genetic disruption [3].

Arising from this flexibility, *C. necator* is under intense study as an industrial host organism for sustainable biomanufacturing [4]. First, it is metabolically versatile, capable of heterotrophic growth on different sugars and organic acids, including industrial waste materials like glycerol [5]. Second, *C. necator* can exploit lithotrophic metabolism to convert CO_2_ into useful products, powered by H_2_ via O_2_‐tolerant [NiFe]‐hydrogenases, with potential applications in greenhouse gas consumption and fuel cell development [6]. Third, the bacterium can accumulate PHA as an energy‐rich storage material. PHAs are biodegradable, potentially providing a sustainable alternative to plastic production that bypasses petroleum‐based sources [7]. Moreover, the PHA‐containing granules can be isolated from the bacterial cell, leading to proposals to manufacture immobilised enzymes or enzyme complexes linked to carbonosome nanoparticles via engineered phasin proteins [8, 9].

Challenges remain, however, in particular the need for sufficient understanding of proteome regulation in order to optimise conflicting metabolic goals. For example, a high growth rate is difficult to achieve in combination with high PHA production [10, 11]. In order to improve our understanding of the proteome‐wide response of *C. necator* to nitrogen limitation (a condition known to stimulate PHA expression), we exploited two global protein analysis approaches (Figure 1B). First, expression proteomics records the signal strength of protein and peptide ions analysed in mass spectrometry (MS) experiments to determine the relative abundance of individual proteins under high and low nitrogen growth conditions. Second, thermal proteome profiling (TPP) also uses MS to quantify the relative stability of proteins subjected to a heat gradient, thereby allowing comparison of protein conformation states under different conditions. By comparing protein expression levels and stability/conformation states in cells grown under balanced media and low nitrogen conditions, a molecular description of the relative roles of the two key regulatory mechanisms governing cellular metabolism—control of protein expression, and allosteric regulation—can be obtained. This allows insight into the global response of *C. necator* to nitrogen stress, including the identification of proteins that respond to metabolic insult via one mechanism or the other, findings that would otherwise be undetected when using only one experimental platform.

We found evidence for expression change during growth in different media among central metabolism enzymes and PHA pathway members, broadly consistent with the expected biological response of *C. neactor*. We then looked for proteins showing evidence of conformational change during growth in different media but that did not show expression change. Several interesting examples were identified, including among ribosomal proteins, Cavlin cycle enzymes and the PHA pathway transcription repressor PhaR. This highlights both the utility of the dual proteome analysis approach, and the nature of the global cell response to nutrient stress, which incorporates multiple regulatory mechanisms operating across distinct pathways.

## 2 Experimental Procedures

### 2.1 Growth of Bacterial Cells

*C. necator* cells were grown on LB broth at 30°C, with shaking (200 rpm). 50 mL of J minimal media (JMM) with sodium gluconate as the carbon source was inoculated with the LB culture to an OD of 0.05. This was left shaking at 30°C, 200 rpm for 48 h. For PHA accumulating conditions (‘nitrogen‐limited media’, N‐lim), nitrogen (ammonium chloride) was limited to 0.1 g/L; otherwise, nitrogen concentration was 16 g/L (‘full media’, Full). Upon harvest, pelleted bacteria were washed twice with PBS and frozen at −80°C until further analysis.

### 2.2 PHA Assay

The method of Lageveen [12] was used. Briefly, cells were harvested by centrifugation, washed in phosphate‐buffered saline (PBS), pH 7.4 and lyophilised using a Labconco freeze‐drier (Fischer Scientific). 5–15 mg (dry weight) of lyophilised cells were resuspended in a combination of 2 mL acidified methanol (15% H_2_SO_4_ v/v) and 2 mL of chloroform containing 6 mg/L benzoate methyl ester, and incubated at 100°C for 3 h, followed by an ice bath for 1 min. 1 mL of water was added, the sample vortexed, and the resulting organic phase (bottom layer) filtered through wool. The 3‐hydroxyalkanoic acid methyl esters were analysed by gas chromatography using an Agilent 6890N chromatograph equipped with a HP‐Innowax capillary column and a flame ionisation detector, using the following temperature program: 120°C for 5 min, increase by 3°C/min to 180°C, 180°C for 10 min.

### 2.3 Expression Proteomics

Harvested cells were lysed by resuspension in 10 mL of 8 M urea, 100 mM ammonium bicarbonate, with one minitablet of HALT protease inhibitor, EDTA‐free (Thermo), sonication on ice three times for 5 s. Following centrifugation (10,000 × *g*, 10 min), protein concentration was determined by Bradford assay. 50 µg of sample was transferred to a Protein LoBind tube (Sigma), and 10 mM dithiothreitol (15 min incubation) followed by 20 mM iodoacetic acid (15 min incubation in the dark). Trypsin was added (trypsin: protein, 1 µg:50 µg) and digestion proceeded overnight at 37°C, 350 rpm in a Thermomixer. Following digestion, samples were acidified to a pH of between 2 and 3 using 100% formic acid and desalted using Zip‐Tips (Thermo). Samples were dried in a Speedvac and stored at −80°C until ready for MS analysis.

### 2.4 Thermal Proteome Profiling

Harvested cells were resuspended in 2 mL of PBS, divided into 10 × 100 µL aliquots, and centrifuged at 5000 × *g* for 5 min. 80 µL of the supernatant was discarded, and the divided samples were distributed across a heat gradient of 30°C–100°C using a LongGene A200 gradient thermal cycler for 3 min. Following this, 50 µL of lysis buffer (50 µg/mL lysozyme, 0.8% IGEPAL, 250U/mL benzonase, 1 mM MgCl2, 1 cOmplete, EDTA‐free protease inhibitor cocktail tablet in 10 mL PBS pH 7.4) was added to each sample, the samples were vortexed and allowed to incubate at room temperature while shaking for 20 min. Samples were submitted to two freeze/thaw cycles at −80°C before centrifugation at 5000 × *g* for 5 min. Protein concentration was determined by Bradford assay, and 50 µg was transferred to LoBind tubes. The volume is brought to 20 µL with PBS. Protein clean‐up and digestion were carried out using Sera‐Mag Speed Beads, according to Hughes and coworkers's method [13]. Briefly, a mixture of hydrophilic and hydrophobic Sera‐Mag Speed Beads (ThermoFischer Scientific 4515‐2105‐050250, 6515‐2105‐050250) was mixed (1:1), and 5 µL of bead stock was added to each protein sample. The samples were shaken at 1000 rpm for 15 min, washed three times on a magnetic rack with 80% ethanol before adding 40 µL of digestion buffer (5 mM choloacetamide, 1.25 mM Tris(2‐carboxyethyl) phosphine hydrochloride (TCEP), 1 µg Trypsin/LysC, in 100 mM HEPES pH 8). Samples were digested overnight at 37°C at 1000 rpm. The beads were recovered using the magnetic rack. Samples were dried and resuspended in ddH2O. Each TMT‐10plex reagent was added to its corresponding sample at a molar ratio of (1:1) and shaken for 1 h. The TMT reaction was quenched by adding 4 µL of 5% hydroxylamine, the TMT‐10plex labelled samples were combined in a single tube, and the entire sample was desalted using Evo‐tips (EvoSep), dried and stored at −80°C until ready for MS.

### 2.5 Liquid Chromatography‐Mass Spectrometry

LC‐MS was performed on a Thermo Scientific Q Exactive mass spectrometer connected to a Dionex Ultimate 3000 (RSLCnano) chromatography system. Tryptic peptides were resuspended in 0.1% formic acid. Each sample was loaded onto a fused silica emitter (75 µm ID, pulled using a laser puller (Sutter Instruments P2000)), packed with Reprocil Pur C18 (1.9 µm) reverse phase media and was separated by an increasing acetonitrile gradient over 300 min at a flow rate of 250 nL/min. The mass spectrometer was operated in positive ion mode with a capillary temperature of 250°C, and with a potential of 2300 V applied to the frit. All data were acquired with the mass spectrometer operating in automatic data dependent switching mode. MS1 parameters include resolution (70,000), scan range (350–1400 m/z), AGC target (3e6). MS2 parameters, resolution (35,000), fixed first mass 100 m/z and AGC target (2e5), Isolation window 1.2 m/z, maximum injection time 250 ms. The Q Exactive was set to select the 12 most intense ions prior to MS/MS analysis using HCD.

### 2.6 Mass Spectrometry Data Analysis

The raw data were searched against the *C. necator* (Strain H16) subset of the Uniprot Swissprot database (6614 entries; www.uniprot.org/proteomes/UP000008210) using the Andromeda algorithm from the Maxquant program (release 2.0.3.0) [14] using the isobaric labelling update parameters for TMT 10plex workflows [15].

### 2.7 Experimental Design and Statistical Rationale

Three technical replicates were employed for the expression proteomics experiments, as well as for bacterial dry weight and PHA analysis experiments. The Student's *t*‐test was used to evaluate significance in pairwise comparison experiments. For expression proteomics experiments, this included permutation‐based FDR adjustment using default settings from the Perseus program (FDR = 0.05; s0 = 2) [13].

For thermal proteomics (TPP) experiments, two technical replicates were used. The TPP R package (https://www.bioconductor.org/packages/release/bioc/html/TPP.html) was used to determine melting temperature (*T_m_*) and other parameters for each protein by fitting reporter ion abundance (normalised to the 30°C sample values) across the thermal gradient to a three‐parameter logistic sigmoid model:

$$y\;\left(T\right)=Pl+\left(a-Pl\right)/\left(1+\exp\left(\frac{T-Tm}{b}\right)\right)$$

where *y*(*T*) is the normalised fold‐change at temperature *T*, *Pl* is the lower plateau, *a* is the upper asymptote, *b* is the slope factor and *T_m_* is the curve inflexion point. The strictest default quality control filter settings were chosen per the TPP R package, removing proteins with low signal intensity or excess missing values, minimum *R* ^2^ and maximum plateau range. Proteins whose melting temperature (*T_m_*) differences between experimental treatments were found to be >1 SD of the population mean were considered significant.

## 3 Results

### 3.1 Low Nitrogen Induces PHA Production in *C. necator* H16

In line with many published studies (reviewed in Kosseva and Rusbandi [16]), we confirmed that growth of *C. necator* in low nitrogen medium (molar ratio C:N = 2.7:1) results in a reduction in growth rate (Figure 1C) and in biomass production (>90% reduction; Figure 1D), concomitant with a dramatic (>200‐fold) increase in PHA production (Figure 1E) compared to growth in complete media (C:N = 27:1). This established the validity of our experimental model in terms of demonstrating a metabolic shift in response to low nitrogen. Subsequent experiments focused on the 48 h time point in order to assess the cell status during the stationary phase (i.e., maximal PHA production).

### 3.2 Global Proteomics of *C. necator* in Nitrogen‐Limiting Media

Next, we asked how the protein complement of *C. necator* responds collectively to the growth conditions of full, nutritionally balanced media versus low nitrogen media. The latter is a well‐known inducer of PHA biosynthesis. Three technical replicates for both full and nitrogen‐limited conditions were analysed in parallel. Briefly, cells were lysed, digested using trypsin, followed by sample clean‐up with reverse phase peptide recovery and liquid chromatography‐mass spectrometry (LC‐MS) analysis using Q Exactive mass spectrometry. Relative expression levels were calculated from extracted MS1 ion signals using the MaxQuant program [14]. Overall, 1835 proteins were identified and quantified (Tables S1 and S2). This represents approximately 28% of the 6614 proteins encoded in the reference genome (https://www.uniprot.org/proteomes/UP000008210). The highly diverse metabolic potential of *C. necator* may result in a lower proportion of the available proteome expressed under a given growth condition compared to other bacteria [17, 18]. We used a long LC‐MS gradient (300 min) in an effort to capture as much proteome readout as possible. Our results are technically comparable to other shotgun proteomics studies of *C. necator* employing LC‐MS [19, 20, 21, 22], suggesting that it reasonably reflects the expressed proteome. One recent study using *C. necator* [23] resulted in the successful identification/quantitation of >4000 proteins. This was achieved for a multivariate analysis of chemostat cultures grown at five different growth rates over four different nutritional limit regimes. The group used a similar LC‐MS analytical platform to our study (90 min gradient) and also incorporated a novel feature detection algorithm for the in silico quantitation step [24]. Analysis of our raw MS data by pairwise comparison found that samples grown under the same conditions displayed higher similarity scores than those grown under differing media conditions (Spearman's Rank Correlation Coefficient 0.65–0.90 and 0.55–0.75, respectively), indicating good reproducibility among replicates (Figure 1F). This finding was supported by hierarchical clustering (Figure 1G), which showed that expression of the majority of proteins (>75%) is stable throughout both nutrition regimes, and principal component analysis (Figure 1H), which found that the majority of data variation (∼40%) reflected experimental differences. These findings give confidence that any expression changes recorded in the proteomics analysis likely represented a genuine biological response.

### 3.3 PHA Pathway Proteins and Calvin Cycle Enzymes Show Expression Change Following Growth in Nitrogen‐Limiting Media

**FIGURE 2:**
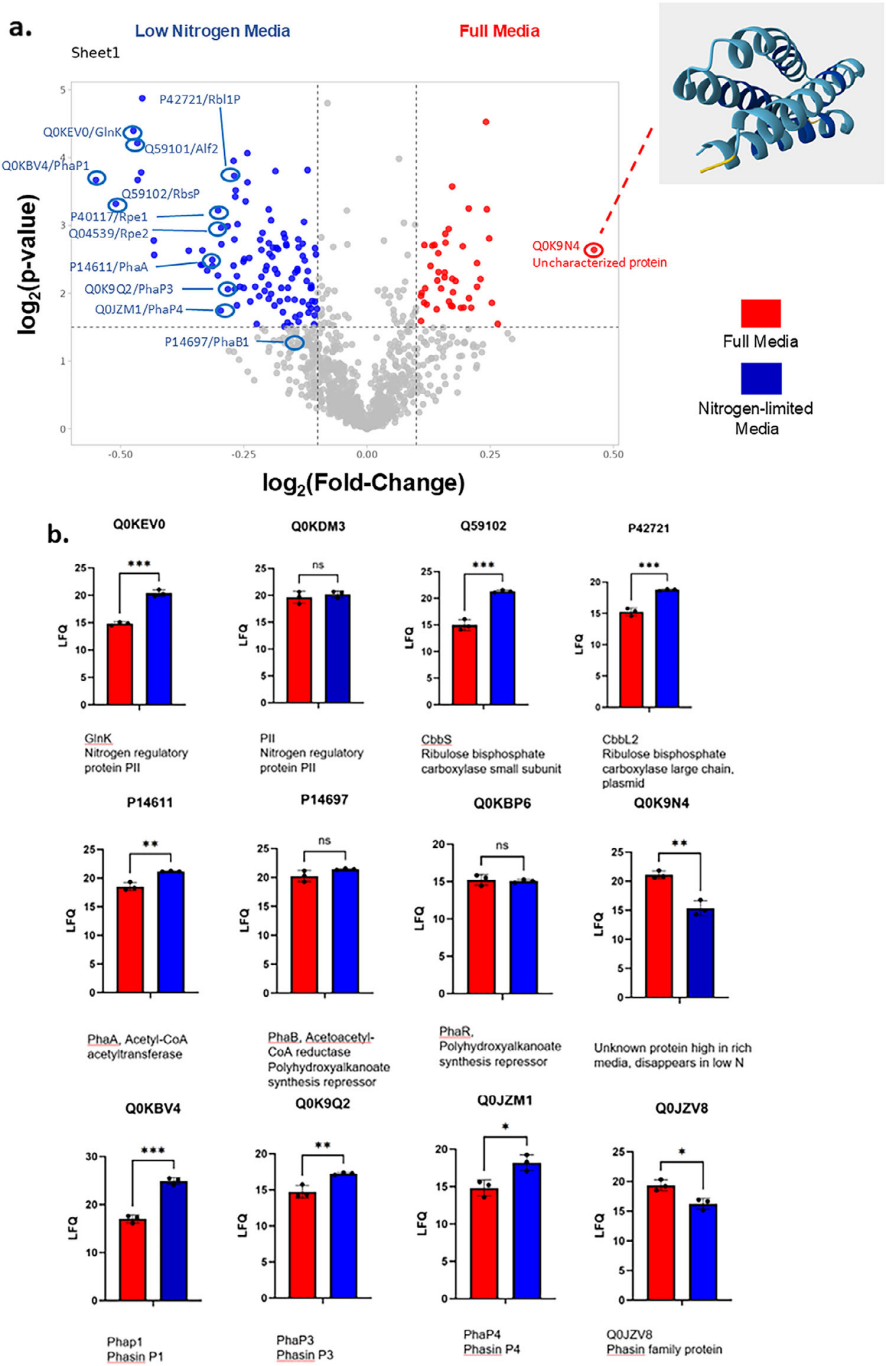
PHA pathway proteins and enzymes of the Calvin cycle are regulated by nutrient stress. (A) Volcano plot comparing the relative expression of individual proteins between growth in balanced media and in low‐nitrogen media. The x‐axis shows the log2(fold change) calculated using LFQ values, and the y‐axis shows the log_2_‐transformed *p* value following FDR processing. The structure for the uncharacterised protein Q0K9N4 predicted by the AlphaFold algorithm is shown in the inset. (B) Plots showing relative abundance values (LFQ, label‐free quantitation) for selected proteins. Comparisons shown are based on the unpaired *t*‐test.

Using the unpaired Student's *t*‐test with cut‐offs of 1.5 (‐log_10_ *p* value) and 0.5 (*t*‐test difference) (Figure 2A), 201 proteins were found to display expression change in one treatment relative to the other. One hundred sixty‐eight proteins showed elevated expression (i.e., were upregulated) under nitrogen‐limited conditions relative to growth in rich media, while 68 showed lower expression levels (Figure 2A; Table S2). Individual proteins were associated with a wide range of functional categories. Enrichment analysis using the DAVID bioinformatics knowledgebase [25] found several significant categories after FDR adjustment, including nine ‘citrate/TCA cycle’, and four proteins containing phasin domains according to the Pfam database (www.ebi.ac.uk/interpro/) (Table S3). Expression of these enzymes and enzyme complexes was upregulated in full media relative low nitrogen conditions, consistent with roles in biomass production during rapid growth [26].

Among the individual proteins demonstrating significantly increased expression in low nitrogen conditions is the nitrogen regulatory protein GlnK, one of two PII signal transduction proteins that play key roles in control of bacterial nitrogen metabolism (Figure 2B). GlnK was also found to be strongly upregulated during a study analysing the growth of *C. necator* on various biodiesel byproducts [21]. We did not observe significant expression change for the PII protein, which is consistent with observations in *Escherichia coli*, where GlnB expression is generally constitutive while GlnK is nitrogen‐responsive [27]. Additionally, both proteins participate in alternate homotrimeric and heterotrimeric complexes, which may also explain our observation [27].

Similarly, individual PHA pathway proteins displayed significant upregulation in low nitrogen conditions (Figure 2B). These included the β‐ketothiolase (PhaA) as well as PHA granule‐associated phasins (PhaP1, PhaP3, PhaP4), and Q0JZV8. The latter is an uncharacterised protein housing a phasin family domain (pfam/PF09361) and predicted by the PONDR‐FIT algorithm to contain an intrinsically disordered region [28]. This is consistent with many studies whereby stress conditions, including low environmental nitrogen levels, have been shown to induce PHA production and PHA granule formation (reviewed in 29). Interestingly, neither PhaB, which catalyses the next‐to‐final step of PHB synthesis, nor PhaR, a negative regulator of phasin expression, demonstrated significant expression change in our experiment (Figure 2B). This raises the possibility that entry to the PHA pathway (i.e., acetyl‐CoA condensation) and granule formation are transcriptionally responsive, while later steps catalysed by PhaB (and PhaC) are constitutively expressed or regulated post‐translationally.

Key enzymes associated with the Calvin (Calvin‐Benson‐Bassham) cycle are also upregulated under nitrogen‐stress conditions. In particular, both CbbS and CbbL2 (respectively the small and large subunits of the Rubisco (Ribulose‐1, 5, bisphosphate carboxylate/oxygenase) carbon fixing complex) show increased expression (Figure 2B). Other significantly upregulated members of the same pathways included ribulose‐phosphate 3‐epimerase (Rpe1, Rep2), fructose‐bisphosphate aldolase (CbbAP), phosphoglycerate kinase (CbbKC) and glyceraldehyde‐3‐phosphate dehydrogenase (CbbGP) (Table S2). This coordinated upregulation suggests that under nitrogen limitation, *C. necator* may activate Calvin cycle enzymes not only for autotrophic growth, but as part of a broad metabolic strategy to balance carbon flux. Interestingly, Jahn and coworkers [23] combined a comprehensive proteomics study with resource balance analysis modelling and concluded that upregulation of Calvin cycle enzymes most likely reflected a need for *C. necator* to maintain a reserve enzyme complement poised for activity should the environmental conditions rapidly change, rather than a route for CO_2_‐reassimilation through Rubisco.

Finally, the previously uncharacterised protein Q0K9N4 is notable for being the protein in our study showing the strongest down‐regulation under low nitrogen conditions (Figure 2B). Sequence queries using various domain mapping software applications (Pfam, Prosite, Interpro) found no evidence of annotated structural domains; however, the AlphaFold algorithm predicts four antiparallel alpha‐helices (https://alphafold.ebi.ac.uk/entry/Q0K9N4; Figure 2A, inset). Furthermore, the protein is conserved among several related *Cupriavidus* species, also among the family Burkholderiaceae. Since it is highly abundant under balanced growth conditions, yet disappears under nitrogen stress, this protein merits further study.

### 3.4 Thermal Proteomics of the *C. necator* Response to Low Nitrogen

**FIGURE 3:**
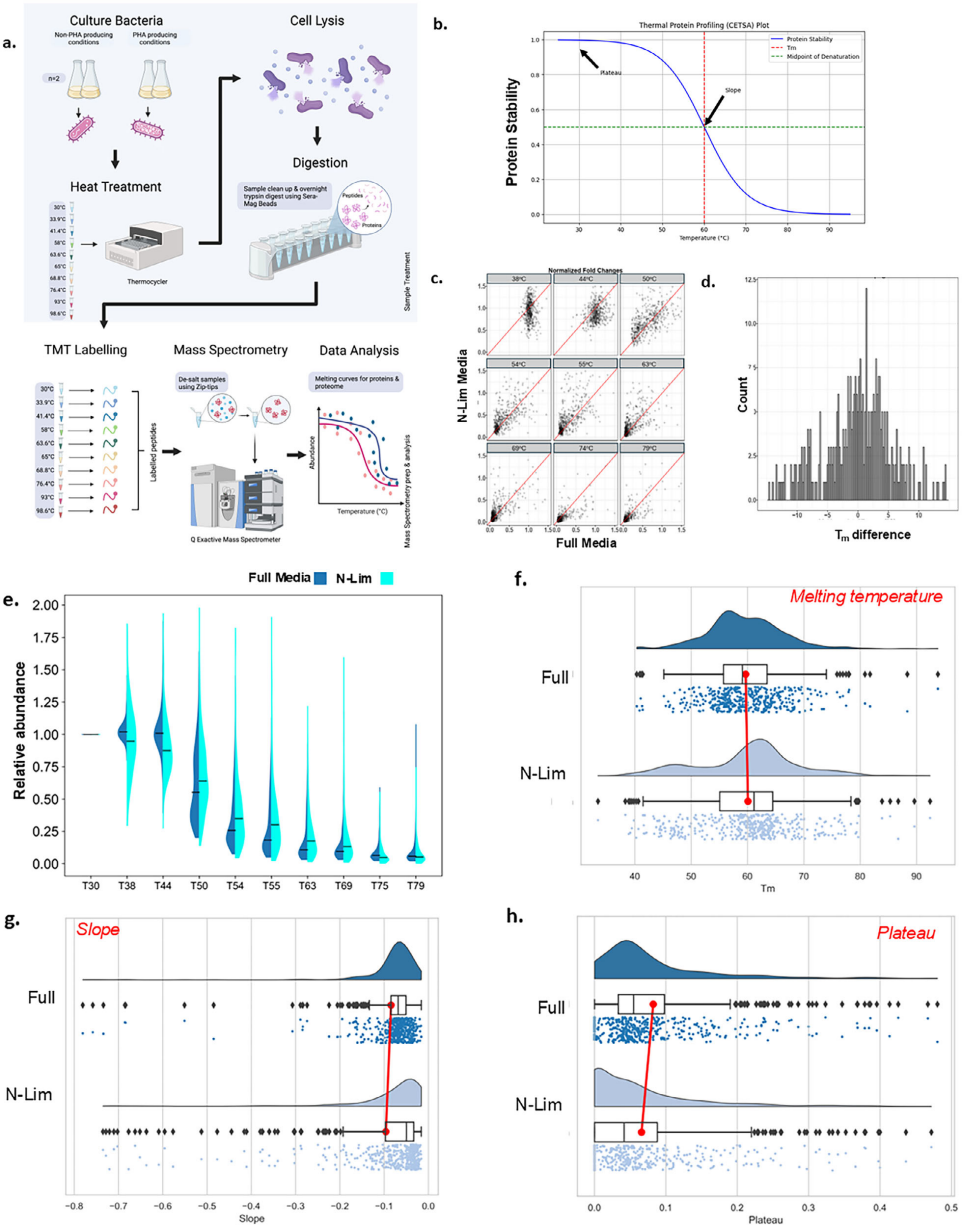
Proteome‐wide analysis of conformation change using thermal proteome profiling (TPP). (A) Parallel *C. necator* cultures (*n* = 2) were grown in full media (PHA‐producing) or limited nitrogen (PHA‐non‐producing) conditions. The intact cells were subjected to a thermal gradient (3 min; 30°C–98°C), following which the cells were lysed, and the heat‐aggregated protein fraction at each temperature point was removed. The samples were digested with trypsin, temperature points encoded with isobaric TMT labelling, and the resulting peptides analysed by Q Exactive mass spectrometry (Thermo Exploris). (B) TPP implements the cellular thermal shift assay (CETSA) at proteome scale. A plot depicting protein loss to denaturation/aggregation over an increasing temperature gradient is produced for each protein. Key parameters that can be compared between experimental treatments include the melting temperature (*T_m_*), the slope of the gradient, and the pre‐ and post‐gradient plateau values. These parameters reflect different aspects of the underlying protein stability/conformational properties. (C) Progressive protein loss to denaturation/aggregation was compared for the two experimental treatments over the gradient temperature steps. (D) Density plot of *T_m_* differences recorded for individual proteins between the two growth conditions using the TPP Bioconductor R package. (E) Relative abundance distribution (normalised to the lowest temperature point) of proteins from cells grown in full or nitrogen‐limited media at each temperature point. (F–H) Comparison of the distribution of *T_m_* (Panel F), slope (Panel G) and post‐gradient plateau (Panel H) parameter values for both experimental treatments.

While bacterial cells often respond to metabolic demands by upregulating the expression of the relevant enzymes (either by transcription or translation control, or both), allosteric regulation (i.e., conformational change to influence enzyme activity induced by binding to sites distal to the active site) is an equally important mechanism [30]. However, this major mode of regulation cannot be detected by conventional quantitative proteomics approaches. We used the thermal proteome profile (TPP) method, which extends the concept of cellular thermal shift assay (CETSA) to the proteome level through the use of isobaric peptide labelling [31] to ask which proteins showed evidence of conformational change in response to low nitrogen growth conditions. Briefly, the intact cells were subjected to a heat gradient, and the conformational change/unfolding response of individual proteins was monitored by the kinetics of protein loss to denaturation or aggregation (Figure 3A) (Table S4). A number of parameters reflecting protein stability under different conditions can be determined in a single TPP experiment (Figure 3B), notably the melting temperature (*T_m_*), the temperature point at which 50% of the protein is present in soluble form. This parameter reflects the thermal stability of an individual protein under different experimental conditions. Similarly, the slope of the transition region of the melting curve reflects the process of unfolding, with a steep slope indicating a rapid, cooperative conformational transition, often typical of compact, well‐folded proteins. The post‐transition plateau reflects the progress of the unfolding process, with a stable, lower plateau indicative of a completely unfolded protein.

In our experiment, both protein populations (i.e., proteins from cells grown in full media or proteins from cells grown in nitrogen‐limited media) were found to progressively aggregate between 30°C and 98°C (Figure 3C).  Four hundred twenty‐eight proteins yielded data that passed the strict quality control criteria of the TPP analysis R package [32], allowing direct comparison of protein stability parameters between the two samples (Table S5). For these proteins, the distribution of melting points between the two treatments was noticeably wide (Figure 3D), confirming that a subset of proteins within the measurable ‘foldome’ was conformationally perturbed in either of the two conditions of the experiment. When scrutinised in more detail, proteins from cells grown under nitrogen‐limited conditions appeared to be, on average, less stable at lower temperature points (38–44°C) than the corresponding proteins from full media cultures, while this trend was reversed at mid temperature points (50°C–69°C) (Figure 3E). There was also more pronounced variation in relative stability among proteins from the nitrogen‐limited media samples, especially at lower temperature points. The reason for these observations is unclear, but they may reflect a more widespread conformational response among proteins adapting to low nitrogen conditions compared to the optimal growth environment of full nutrient availability. While we are unaware of studies addressing this at proteome‐wide level, there are several specific examples of allosteric or post‐translational modification changes in response to nitrogen metabolism, for example, PII proteins, NtrC family regulators, glutamine synthase [33, 34, 35]. No significant differences were found between the two treatments for the parameters of melting temperature or slope (unpaired *t*‐test; *p* > 0.05) (Figure 3F,G), while average post‐transition plateau values were found to be lower for proteins of the nitrogen‐limited sample (*p* = 0.0044) (Figure 3H), although the effect was modest (mean for full media proteins = 0.08; mean for nitrogen‐limited proteins = 0.06). These results, showing that the majority of proteins are conformationally unperturbed, are in agreement with recent large‐scale studies in *E. coli, Geobacillus stearothermophilus* and *Thermus thermophilus* [36, 37].

### 3.5 Biophysical Properties of Stabilised and Destabilised Proteins

**FIGURE 4:**
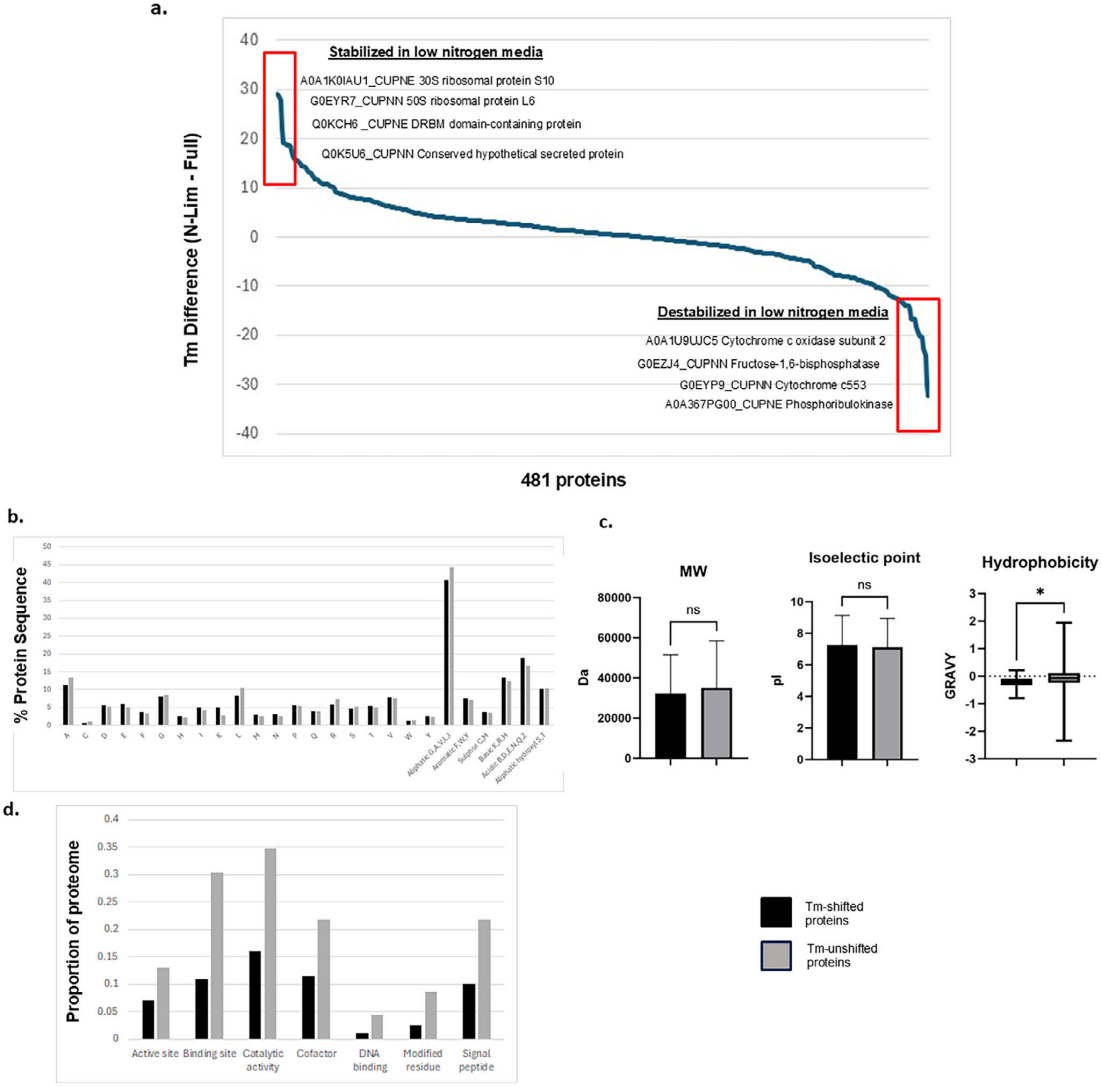
TPP global analysis. (A) Ranked distribution plot of *T_m_* differences observed between *C. necator* proteins grown in full or in low‐nitrogen media. Top‐ranking proteins with elevated *T_m_* values in low nitrogen conditions (i.e., conformationally stabilised in response to nutrient stress) are shown in box on left; top‐ranking proteins with decreased *T_m_* values (i.e., destabilised in response to nutrient stress) are shown in box on right. (B) Comparison of amino acid composition between *T_m_* shifted and *T_m_* unshifted proteins. (C) Comparison of MW, pI, and hydrophobicity (GRAVY index) between *T_m_* shifted and *T_m_* unshifted proteins. (D) Frequency of keywords describing *T_m_* shifted and *T_m_* unshifted proteins (from the Uniprot database).

We defined proteins showing evidence of conformational change in response to nitrogen limitation conditions as those displaying *T_m_* change (between full and nitrogen‐limited growth) of greater than 1 SD (standard deviation) beyond the average global *T_m_* difference (Figure 4A; Table S5). Thirty‐six proteins met this criteria; 20 showing evidence of stabilisation (*T_m_* is higher following growth in full media) and 16 showing evidence of destabilisation (*T_m_* is lower following growth in nitrogen‐limited media). Many ribosomal proteins were ranked highly as stabilised proteins, as well as a protein of unknown function containing a DRBM (dsRNA‐binding) domain, Q0KCH6, and the ‘conserved hypothetical secreted protein’ Q0K5U6. In contrast, proteins found to rank highly as destabilised in low nitrogen growth included well characterised central metabolism enzymes in involved in diverse pathways such as phosphoribulokinase (Calvin cycle), cytochrome c553 (redox balance, energy metabolism), fructose‐1,6 bisphosphatase (Calvin cycle, gluconeogenesis) and cytochrome c oxidase subunit 2 (respiratory electron transport chain). These observations of preferential stabilisation of ribosomal proteins and destabilisation of central metabolism enzymes mirror large‐scale studies in other bacteria [36, 37]. Stabilisation of ribosome proteins potentially reflects hibernation or protective complex formation [38], while reduced stability of enzymes involved in central metabolism in thermal proteomics experiments may reflect physical interactions with metabolites and/or binding proteins [39].

We examined the broad biophysical properties of the proteins found in these sets. No major differences were observed in amino acid content between *T_m_*‐shifted and *T_m_*‐unshifted proteins, apart from a modest decrease in aliphatic, mainly hydrophobic amino acids (A, G, I, L, V) and a modest increase in both acidic (H, K) and basic (E, D) amino acids among *T_m_*‐shifted proteins (Figure 4B). Similarly, no significant differences were noted for either MW or pI between the sets, while *T_m_*‐shifted proteins displayed lower hydrophobicity according to the GRAVY index [40] (Figure 4C). These observations could plausibly reflect conformation effects since the presence of both hydrophobic and charged residues is linked to protein stability [41]. Using keyword annotations collected from the Uniprot database, *T_m_*‐shifted proteins also appeared to be less likely than their unshifted counterparts to contain signal peptides, enzyme active sites, binding sites, modified residues, catalytic activity, or be associated with DNA‐binding or cofactor activity (Figure 4D). We found no evidence that *T_m_*‐shifted proteins differed as a group from non‐*T_m_*‐shifted proteins in the frequency of intrinsically disordered regions (Fisher's Exact Test, *p* = 0.6044; DisProt database) [42].

### 3.6 Ribosomal Proteins Are Stabilised, and Proteins of the Calvin Cycle Destabilised, in Response to Nutrition Stress

**FIGURE 5:**
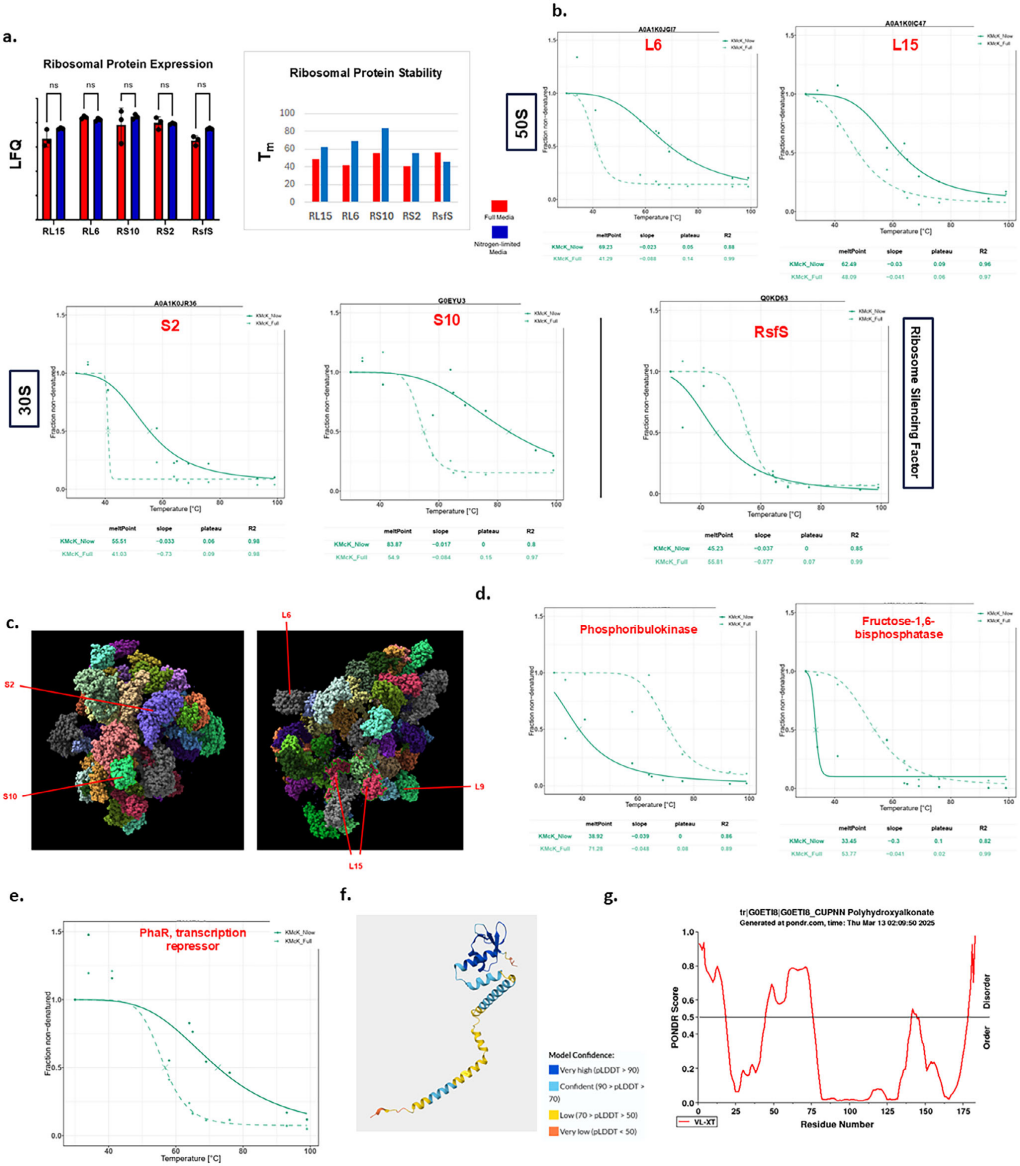
Stability/conformation behaviour of selected proteins. (A) Several ribosome proteins show significant stability change between full media and low‐nitrogen media growth conditions (right panel), despite showing no significant expression change between these states (left panel). The ribosome silencing factor RsfS demonstrates the opposite trend, being destabilised in full media conditions. (B) Thermal stability profiles for ribosomal 50S (L6, L15) and 30S (S2, S10) subunit proteins show a significant *T_m_* shift between experimental treatments in the TPP experiment. (C) Ribosomal proteins discussed in the text were mapped to the *Pseudomonas aeruginosa* reference ribosome structure. (D) Comparative thermal stability profiles for Calvin cycle enzymes were analysed following growth under full or low‐nitrogen conditions. (E) Comparative thermal stability profiles for the PHA pathway transcription regulator PhaR following growth under full or low‐nitrogen conditions. (F) Predicted structure of the PhaR protein produced using AlphaFold. (G) Protein disorder prediction for PhaR was produced using the PONDR algorithm.

Ribosomal proteins were notably prominent in the list of high‐ranking proteins stabilised under nitrogen‐limiting conditions despite showing no evidence of expression change between the growth conditions (Figure 5A; Table S5). Five proteins were found to show significant melting point change between the two conditions, three from the large (50S) ribosomal subunit (RL6, RL9, RL15), and two from the small (30S) subunit (RS2, RS10) (Figure 5B). When mapped to the *Pseudomonas aeruginosa* ribosome reference map (https://www.rcsb.org/structure/8rwg), these proteins were found to be distributed widely around the structure (Figure 5C), showing neither obvious structural proximity nor functional links [43]. However, we found that the ribosomal silencing factor, RsfS, shows the opposite trend to the component ribosome proteins, being destabilised under low‐nitrogen growth conditions. Studies in several bacterial species show that RsfS is linked to reducing translation levels during the transition from rich to poor media, and likely mediates this function by inhibiting the formation of productive 70S ribosomal complexes by preventing 50S and 30S subunit association [44, 45]. This supports the widespread evidence that ribosomes are subject to multiple levels of molecular control in response to growth conditions, including hibernation and the regulation of assembly and interaction with ancillary factors [38, 46].

Two proteins prominent among those found to show greatest destabilisation in nitrogen‐limiting growth conditions are phosphoribulokinase (PRK; ranked 1) and fructose‐1,6‐bisphosphatase (FBP; ranked 5) (Figure 5D). These enzymes operate at key steps of the Calvin cycle and both are allosterically regulated [47, 48], yet neither was among the proteins found to change expression levels in response to low nitrogen conditions in our expression proteomics experiments (Figure 2A). This underscores the roles of enzymes like PRK and FBP as integrators of multiple regulatory inputs, including metabolite binding, redox state, and conformational switching, to balance carbon fixation with broader demand of nitrogen‐limited metabolism [49].

Finally, we found that the PHA pathway transcriptional repressor PhaR was more thermally stable in bacteria grown in nitrogen‐limiting conditions (Figure 5E), despite showing no significant expression change between growth states. In *C. necator* and other PHA‐producing bacteria, PhaR regulates PHB metabolism by binding the promoter of its own gene, and to the phasin operon *phaP* [50, 51]. The N‐terminus of PhaR contains a ‘PHB/PHA accumulation regulator DNA‐binding’ domain (Pfam: PF07879) that is believed to house the DNA‐binding activity since it is highly conserved among PHA‐producing bacteria (https://www.ebi.ac.uk/interpro/entry/pfam/PF07879/), while a central ‘PHB accumulation regulatory’ domain (Pfam: PF05233) is believed to house a PHB‐associating portion of the protein (Figure 5F). Interestingly, the PONDR algorithm predicts a disordered region at the N‐terminus of PhaR (Figure 5G), and coincides with a low confidence portion of the Alpha‐Fold structural prediction, raising the possibility that this region may be a candidate for conformational change in response to nutrition stress. Previous studies employing PhaR in engineered regulatory systems and biosensors demonstrate that PHB‐bound PhaR is conformationally distinct, stably associated with hydrophobic surfaces, and protected from proteolysis [52]. Collectively, these findings support the interpretation that the increased stability of PhaR under nitrogen limitation reflects its transition from a DNA‐bound to a PHB‐bound state.

## 4 Discussion

*C. necator*, formerly known as *Ralstonia eutropha*, has been flagged for many years as a promising vehicle for many applications in biotechnology [53]. These include carbon capture and bioplastic production, biodegradation of pollutants, production of biohydrogen, microbial electrolysis cells, and synthetic biology applications such as protein display on PHA granules. *C. necator* can also fix CO_2_ via a functional Calvin cycle, potentially contributing to carbon capture technologies and the conversion of greenhouse emissions into bioplastics or biofuels. The marked metabolic versatility of this organism is countered by a trade‐off between conditions that are optimal for cell growth and those that promote PHA biosynthesis [10, 11]. This makes it challenging to combine high growth rates with high levels of PHA production, limiting the use of *C. necator* as an agent for sustainable biotechnology. While this work is not the first to investigate the response to *C. necator* to nutrient stress at the proteome level, to our knowledge, it is the first to also incorporate a thermal proteomics analysis.

In the present study, we found evidence for proteins that respond to stressful nutrient environments through two main mechanisms: protein expression change and protein stability (i.e., conformation) change. In order to understand these processes in more detail, we adapted two MS‐based approaches that provide insight into both mechanisms: quantitative proteomics (regulation of protein expression) and TPP (protein conformation change). Many of our observations are understandable in terms of previous knowledge of *C. necator*. For example, PHB production is well known to be upregulated under stress conditions, presumably to provide energy resources for challenging or uncertain future environments, and we observed the up‐regulation of several PHA pathway proteins and phasins. However, we also observed upregulation of Calvin cycle enzymes, as well as evidence for stability changes among other enzymes within the same pathways. This is unexpected since this pathway is associated with growth under lithoautotrophic conditions. Interestingly, a recent study using LC‐MS and resource balance analysis found that *C. necator* produces proteins in excess of the minimal requirements under many metabolic states that were modelled [23]. While this likely represents an adaptation to the wild state where the bacterium may be required to switch metabolic modes in response to rapidly changing environmental conditions, it can represent a limiting factor for industrial applications that to date has not been overcome. More precise modelling based on an improved understanding of the *C. necator* protein network may facilitate these efforts. For example, we report a protein that is prominently expressed during growth in rich media but that effectively disappears in low growth conditions (Q0K9N4). There was insufficient data from the TPP experiment to compare the stability/conformation behaviour of this protein between the experimental states. However, the expression characteristics open the possibility that it has effector or regulatory properties related to nutrition/stress response, and it will be an interesting subject of further study since its function is unknown. Our results are broadly consistent with findings in other bacteria, for example, *E. coli* and *Bacillus subtilis*, where proteome‐scale studies have also identified partially overlapping sets of proteins responding to nutrient stress by expression change and/or conformation change [36, 37].

The TPP experiments identified sets of *C. necator* proteins that respond to low nitrogen conditions through either increased stabilisation or increased destabilisation. We found examples among ribosomal proteins, Calvin cycle enzymes, and the PhaR transcription regulator. Under balanced growth conditions, PhaR binds the promoter of the phasin PhaP1 and the intergenic region of phasin PhaP3, thereby inhibiting their transcription, but upon induction of PHA granule production, it associates with the granule, thus reversing the repressing effect [54, 55]. This raises the possibility that PhaR undergoes a conformation change during nutrient stress that alters its affinity from *pha* operon DNA sequences toward the PHA granule environment.

While there can be many potential explanations of our observations, stabilisation often indicates that a protein has adopted a conformation suitable for optimal enzymatic or binding activity under the tested condition. Conversely, destabilisation can indicate that the protein is destined for elimination, or that it has adapted its conformation to engage with another protein, substrate or ligand, in response to the tested condition. Regardless, both situations provide evidence that the protein is responding to the tested condition. Particularly, the case of proteins that otherwise do not show expression change (as is the case for PhaR and the Calvin cycle and ribosomal proteins discussed here), this can indicate an allosteric response. A limitation of the current study is that these factors are not directly addressed—using expression proteomics or TPP, the local structural behaviour of the protein (e.g., instability of protein domains or destabilising individual amino acids) is not observed, nor the differential addition or removal of post‐translational modifications. Both these factors could be investigated in future studies through the use of techniques such as limited proteolysis, cross‐linking or deuterium exchange studies, or enrichment for specific protein modifications coupled to targeted MS. Additionally, our study was based on the use of technical rather than biological replicates, limiting our understanding of the influence of biological variation on the findings.

Taken together, our findings support a model in which nitrogen‐limitation in *C. necator* elicits a dual‐layer proteome response: (a) transcription/translation changes that remodel metabolic capacity, and (b) abundance‐independent conformation changes in key metabolic and regulatory proteins. The latter includes stabilisation of ribosomal proteins, perhaps consistent with hibernation, conformation shifts in Calvin cycle proteins plausibly linked to altered redox status and metabolite levels, and PHB‐binding stabilisation of the transcript regulator PhaR. These effects potentially enable rapid, reversible adjustment of metabolic activity, a key requirement of a metabolically versatile organism like *C. necator*. Aspects of these models are testable. PhaR structure could be investigated using techniques like cross‐link MS under PHA‐inducing or non‐inducing conditions to test for conformation change. The apparent destabilisation of Calvin cycle enzymes could be investigated by similar means, in addition to measuring the kinetic parameters of enzymes isolated under nitrogen‐limiting or full media conditions. Similarly, evidence for ribosome hibernation under nitrogen‐limiting conditions, for example, the presence of 100S complexes, could be investigated. Finally, while we focused on a single stress condition (low available nitrogen), a broader study that included other stresses (i.e., other nutrient limitations as well as redox, osmotic, etc.) may be informative in terms of whether the responses are specific or generalised.

## Author Contributions

G.C. and K.McK. designed the study. K.McK. carried out the majority of the experimental work. J.L.T., M.B., T.N. and K.O.C. assisted with experimental work. K.W. and E.D. carried out the mass spectrometry experiments. G.C., K.W. and K.McK. carried out the data analysis. G.C. wrote the manuscript with assistance from K.McK. G.C. and K.O.C. were involved in funding acquisition. All authors reviewed the manuscript.

## Conflicts of Interest

The authors declare no conflicts of interest.

## Supporting information




**Supporting File**: pmic70045‐sup‐0001‐Tables.zip.

## Data Availability

The mass spectrometry proteomics data have been deposited to the ProteomeXchange Consortium via the PRIDE partner repository with the dataset identifier PXD062592.
